# Flcn-deficient renal cells are tumorigenic and sensitive to mTOR suppression

**DOI:** 10.18632/oncotarget.5018

**Published:** 2015-09-21

**Authors:** Mingsong Wu, Shuhui Si, Yan Li, Susan Schoen, Guang-Qian Xiao, Xueying Li, Bin Tean Teh, Guan Wu, Jindong Chen

**Affiliations:** ^1^ Department of Cell Biology and Genetics, Zunyi Medical University, Zunyi 563099, China; ^2^ Kidney Cancer Research Laboratory, Department of Urology, University of Rochester Medical Center, Rochester, NY 14642, USA; ^3^ State Key Laboratory of Bioactive Substances and Functions of Nature Medicines, Institute of Materia Medica, Chinese Academy of Medical Sciences & Peking Union Medical College, Beijing 100050, China; ^4^ Department of Pathology, University of Rochester Medical Center, Rochester, NY 14642, USA; ^5^ NCCS-VARI Translational Cancer Research Laboratory, National Cancer Centre, 169610, Singapore

**Keywords:** BHD, RCC, kidney cancer, mTOR, sirolimus

## Abstract

Deficiency of tumor suppressor FLCN leads to the activation of the mTOR signaling pathway in human BHD-associated renal cell carcinomas (RCC). We have previously developed a renal distal tubule-collecting duct-Henle's loop-specific *Flcn* knockout (KO) mouse model (*Flcn*^flox/flox^/*Ksp-Cre*). This mouse model can only survive for three weeks after birth due to the development of polycystic kidney and uremia. Whether these cystic solid hyperplasia changes seen in those KO mice are tumorigenic or malignant is unknown. In this study, we demonstrated that genetic disruption of *Flcn* in mouse kidney distal tubule cells could lead to tumorigenic transformation of these cells to develop allograft tumors with an aggressive histologic phenotype. Consistent with previous reports, we showed that the mTOR pathway plays an important role in the growth of these Flcn-deficient allograft and human UOK 257-1 xenograft tumors. We further demonstrated that the mTOR inhibitor, sirolimus, suppresses the tumor's growth, suggesting that mTOR inhibitors might be effective in control of FLCN-deficient RCC, especially in BHD renal tumorigenesis.

## INTRODUCTION

The mammalian target of rapamycin (mTOR) is a protein kinase that integrates a variety of signaling pathways that regulate cellular growth, proliferation and metabolism [1]. The mTOR molecule involves two functionally distinct, but cross-talk complexes, mTOR complex 1 (mTORC1) and mTOR complex 2 (mTORC2) [2]. Targeting mTOR is one of the therapeutic strategies for many mTOR-related tumorigenesis including kidney cancer [3]. A few mTOR inhibitors have been identified in the past decades [4]. Sirolimus, also known as rapamycin, is one of the inhibitors of mTOR [4]. Sirolimus provides its therapeutic effects by inhibiting both protein kinase complex mTORC1 and mTORC2. Sirolimus inhibits the growth of many tumor cell lines *in vitro* and exhibits antitumor activity in murine tumor models [4]. Sirolimus also affects angiogenesis in tumors. Guba et al. demonstrated in a mouse model that sirolimus inhibited tumor progression through antiangiogenic activity related to impaired production of VEGF and limiting proliferative response of endothelial cells to stimulation by VEGF [5]. Luan et al. reported similar findings in a mouse model of metastatic renal cell carcinoma (RCC) [6]. Additionally, sirolimus has also been shown to inhibit the progression of dermal Kaposi's sarcoma [7].

FLCN (folliculin), a tumor suppressor, was originally identified from patients with Birt–Hogg–Dubé (BHD) disease [8]. BHD disease is an inherited kidney cancer syndrome that predisposes patients to develop hair follicle tumors, kidney cancers, lung cysts, and spontaneous pneumothorax [8, 9]. Generally, most of kidney cancers (>90%) are renal cell carcinomas (RCC) that are subtyped histologically as clear cell RCC (70–80%), papillary RCC (10–15%), chromophobe RCC (5–10%), and collecting duct carcinoma (<1%). However, of the BHD-related kidney tumors, the majority are chromophobe RCC and chromophobe RCC/oncocytoma hybrid [10]. In addition, besides BHD, there are a few other kidney cancer-related syndromes such as von Hippel-Lindau (VHL) syndrome [11], hereditary papillary renal carcinoma type 1 (HPRC) hereditary leiomyomatosis [12] renal cell cancer (HLRCC), and tuberous sclerosis (TS) [13]. All of the syndromes are genotype-specific, namely, VHL, HPRC, HLRCC, TS, and BHD are caused by mutated *VHL*, *c-Met*, *FH*, *TSC/TSC2*, and *FLCN*, respectively. Interestingly, while each of the above syndromes is subtype-specific, BHD syndrome predisposes to developing all subtypes of RCCs 10, which indicates that these syndromes have diverse genetic and pathogenic backgrounds in renal tumorigenesis 14, and only FLCN plays a role of universal renal tumor suppressor. In addition, FLCN mutations and loss of its mRNA expression have been observed in various types of sporadic tumors including colorectal cancer, endometrial carcinoma, gastric cancer, and other types of tumors developed in BHD patients [8, 15–27], which further suggests that FLCN is a universal cancer suppressor. *In vitro* cell experiments and *in vivo Flcn* knockout mouse model studies indicated that loss of FLCN led to the activation of the mTOR pathway [28–34]. These findings suggest that up-regulation of mTOR pathway is involved in BHD tumorigenesis and mTOR could be an effective drug target for FLCN-deficient tumorigenesis.

In our previous study, we have developed a renal distal tubule-collecting duct-Henle's loop-specific *Flcn* knockout (KO) mouse model (*Flcn*^flox/flox^/*Ksp-Cre*) [31]. This mouse model can only survive three weeks after birth due to the development of polycystic kidneys and uremia. Whether the cystic solid hyperplasia seen in these KO mice is tumorigenic or malignant is unknown. In this study, we demonstrated that cells isolated from the mouse Flcn-deficient renal cysts containing cystic solid hyperplasia could evolve into malignant tumorigenic cells *in vitro*, and could develop allograft tumors after being injected into nude mice. Moreover, we further developed a xenograft tumor mouse model by inoculating human FLCN-deficient UOK 257-1 cells [35]. Since no therapeutic approaches for BHD kidney tumorigenesis have been described, targeting the mTOR pathway could be an effective therapeutic strategy for BHD-specific or FLCN-deficient kidney tumorigenesis. Herein, we treated these allograft and xenograft mouse models with the mTOR inhibitor, sirolimus, and demonstrated that sirolimus could efficiently suppress FLCN-deficient tumor growth.

## RESULTS

### Flcn-deficient renal distal tubule cells evolved into aggressive tumor cells and allograft tumors

**Figure 1:**
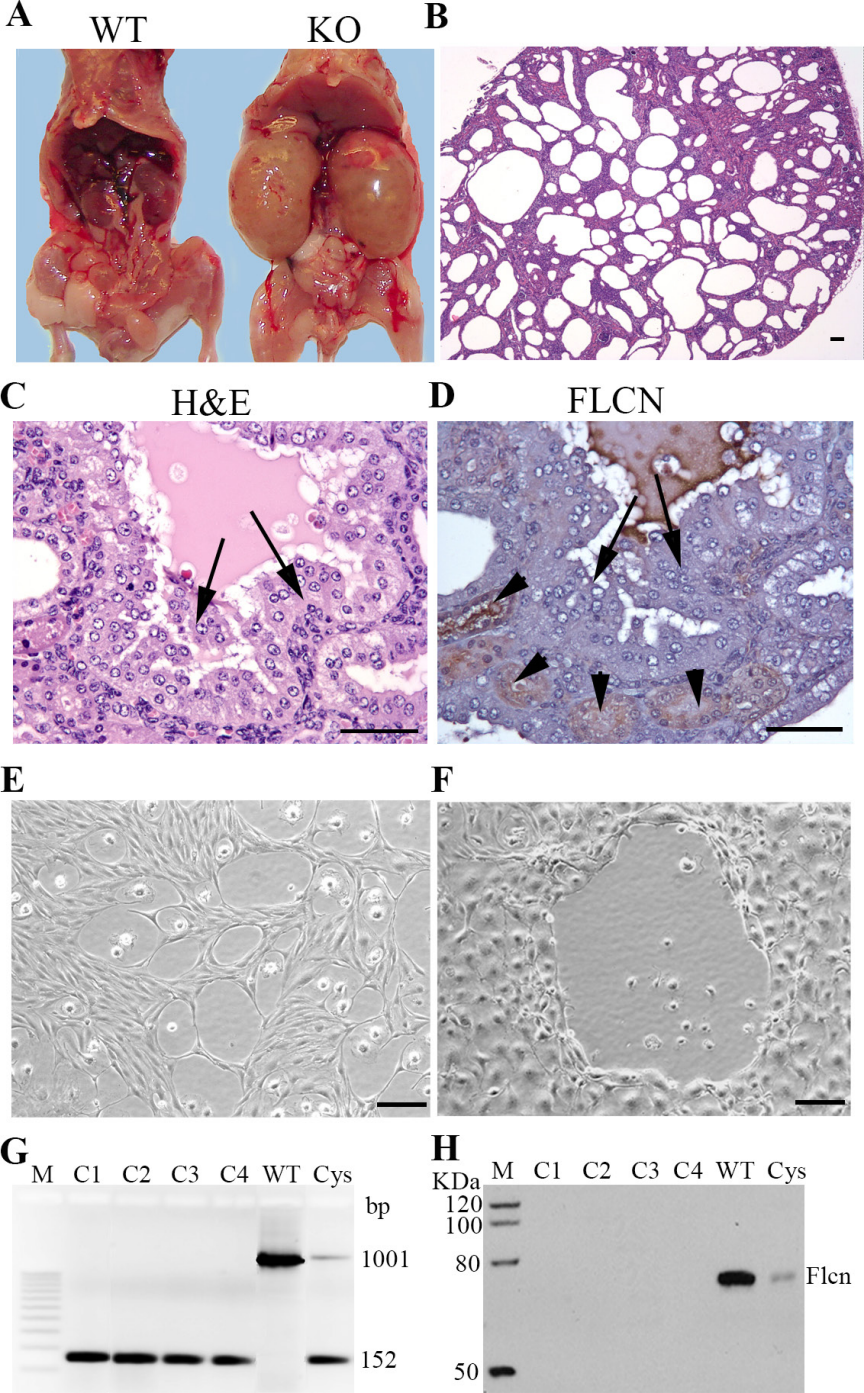
Generation of tumorigenic Flcn-deficient renal distal tubule cell lines **A.** phenotype of the KO (*Flcn^flox/flox^/Ksp-Cre* knockout) mice. KO kidneys were enlarged due to polycystic changes compared to WT ones. **B.** H&E staining of the polycystic kidneys of *Flcn^flox/flox^/Ksp-Cre* mice at age of 10 days. **C.** hyperplasia/micro-tumors identified in a *Flcn^flox/flox^/Ksp-Cre* mouse kidney (indicated by arrows). **D.** No Flcn expression observed in the hyperplasia/micro-tumors (indicated by arrows). Note that the hyperplasia/micro-tumors were Flcn negative compared to the proximal tubules stained positively (indicated by arrow heads). **E, F.** representative cells lines isolated from two *Flcn^flox/flox^/Ksp-Cre* polycystic/micro-tumor kidneys and cultured in DMEM medium. **G.** PCR genotyping demonstrated that cell lines derived from four KO kidneys (C1-C4) displayed KO band (152 bp), indicated that *Flcn* had been disrupted. Wild-type kidney (*Flcn^flox/flox^*) showed only a wild-type band (1001 bp). Cystic kidney (Cys) had both wild-type band and KO band due to the mixture of *Flcn* disrupted and undisrupted renal cells. **H.** Western blot analysis demonstrated that the cells (C1–C4) have no Flcn expression. Cystic kidney tissue showed weak Flcn expression. WT, wild type; KO, knockout. Cys, cystic kidney. Bar scale, 50 μm.

To determine whether genetic disruption of *Flcn* in mouse kidney distal tubule cells can lead to development of kidney neoplasm, we have previously generated distal tubule-collecting duct-specific knockout mice by breeding *Flcn^flox/flox^* mice to *Ksp-Cre* transgenic mice with expression of *Cre-recombinase* under the control of the *kidney-specific cadherin promoter* [31]. No substantial solid tumors other than cysts and solid hyperplasia were observed in all the affected mice (Figure 1A–1C), which is likely due to the short lifespan of the *Flcn^flox/flox^/Ksp-Cre* mice due to polycystic changes of the kidneys and uremia. Thus, in this study, we isolated and cultivated cells from the cystic hyperplasia and micro-tumors of kidney-specific homozygous knockout mice *Flcn*^flox/flox^/*Ksp-Cre*, and inoculated them into athymic nude mice to see whether tumors would develop in these hosts.

**Figure 2:**
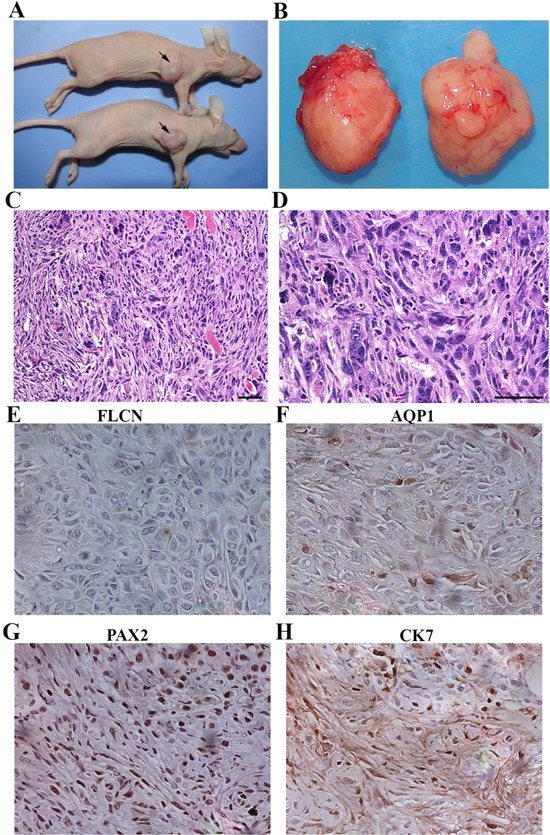
Flcn-deficient renal distal tubule cells are tumorigenic **A.** inoculation of Flcn-deficient renal distal tubule cells developed into allograft tumors in nude mice. **B.** allograft tumors obtained in the nude mice. **C, D.** H&E staining of these tumor tissue sections revealed that all tumors were high-grade renal cell carcinomas (sarcomatoid RCC). **E.** IHC analysis with FLCN antibody showed no Flcn expression in the allograft tumors. **F, G.** and **H.** IHC analysis by renal cell carcinoma makers (AQP1, PAX2, and CK7) revealed that the allograft tumors were positive for these markers, indicating that the allograft tumors were renal cell carcinomas. Bar scale, 50 μm.

The cystic renal cells were isolated from the polycystic kidneys and cultivated *in vitro* for 35 passages or more (Figure 1D). Six *Flcn^flox/flox^/Ksp-Cre* kidneys were used for isolating cystic renal cells. While most of the cells died out, the pre-malignant or malignant cells survived (Figure 1E–1F). Four cell lines were successfully obtained. To determine whether the survived cells are *Flcn*-disrupted, we performed genotyping on the four selected cell lines. All the four cell lines are *Flcn*-deleted (Figure 1G). Western blot analysis further validated the result (Figure 1H). The surviving cells were collected and maintained to establish cell lines. Inoculated subcutaneously in the athymic nude mice, these cells grew into allograft renal tumors after one month (Figure 2A–2B). The control HEK-293 normal kidney cells did not develop any tumors. The Flcn-deficient tumors were histologically composed of mixed spindle cells and epithelial cells with high malignant morphology (Figure 2C–2D). Immunohistochemical analysis with a panel of kidney-specific markers (AQP1, PAX2, and CK7) demonstrated that these tumors were renal cell carcinomas. (Figure 2E–2H) The histological features are consistent with sarcomatoid renal cell carcinoma (SRCC) (Figure 2F–2H). Human SRCC accounts for 1% to 5% of all renal malignant neoplasms [36]. Patients with sarcomatoid tumors usually present with poor prognosis for all stages of the disease. While SRCC may evolve from any type of renal cell carcinoma, many reports have noted that chromophobe cell carcinoma is the most common histologic variant associated with SRCC transformation [37–43]. Correspondingly, 84% of the human kidney tumors from BHD patients were chromophobe or chromophobe/oncocytoma hybrid [10]. Thus, it is not surprising to see that Flcn-deficient renal cystic cells ultimately develop into SRCCs.

### The mTOR pathway was up-regulated in Flcn-deficient allograft tumors

**Figure 3:**
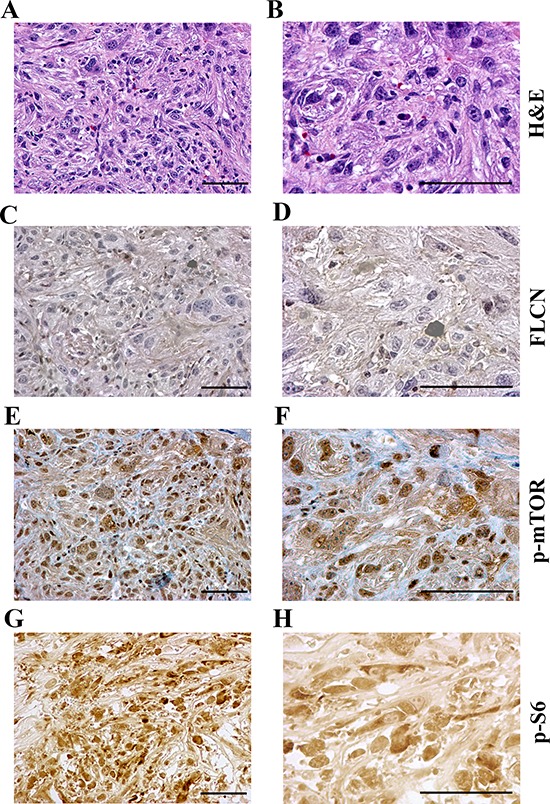
The mTOR pathway is activated in allograft tumors developed from Flcn-deficient renal distal tubule cell lines **A, B.** H&E staining of tumors. **C, D.** Flcn was not expressed in allograft tumor. **E, F.** mTOR was activated by phosphorylation in Flcn-deficient tumor. **G, H.** phosphorylated activation of S6 was also observed in tumors. Bar scale, 50 μm.

To elucidate the biochemical mechanism of the cystogenesis and carcinogenesis related to inactivation of *Flcn* in these allograft tumors, we investigated the possible relevance of Flcn to the mTOR signaling pathway. Since we have demonstrated that Flcn deficiency leads to the activation of the mTOR pathway in those kidney cysts [31], we expected that mTOR was also activated in these high-grade allograft RCCs originated from the cystic hyperplasia/micro-tumor cells. First, we observed that the allograft tumors (Figure 3A–3B) were Flcn negative (Figure 3C–3D), indicating the tumors derived from Flcn-null cystic renal tubule cells. We then further examined whether the inactivation of *Flcn* was associated with the up-regulation of mTOR in the allograft tumors as it does in Flcn-deficient cysts. Immunohistochemical analysis revealed that mTOR was also activated through phosphorylation in allograft sarcomatoid tumors (Figure 3E–3F), which were Flcn-staining negative (Figure 3C–3D). In contrast, the Flcn-positive cells showed negative p-mTOR signals. To gain further insight into the relevance of Flcn to the mTOR pathway, we next examined the phosphorylated status of the downstream targets S6. Phosphorylated S6 has been observed in some of the tumors (Figure 3G–3H), which is consistent with our observation in kidney cysts of *Flcn^flox/flox^/Ksp-Cre* mice. Therefore, these results suggest that Flcn is connected to the mTOR signaling network.

### Sirolimus inhibited mouse allograft Flcn-deficient tumor growth

**Figure 4:**
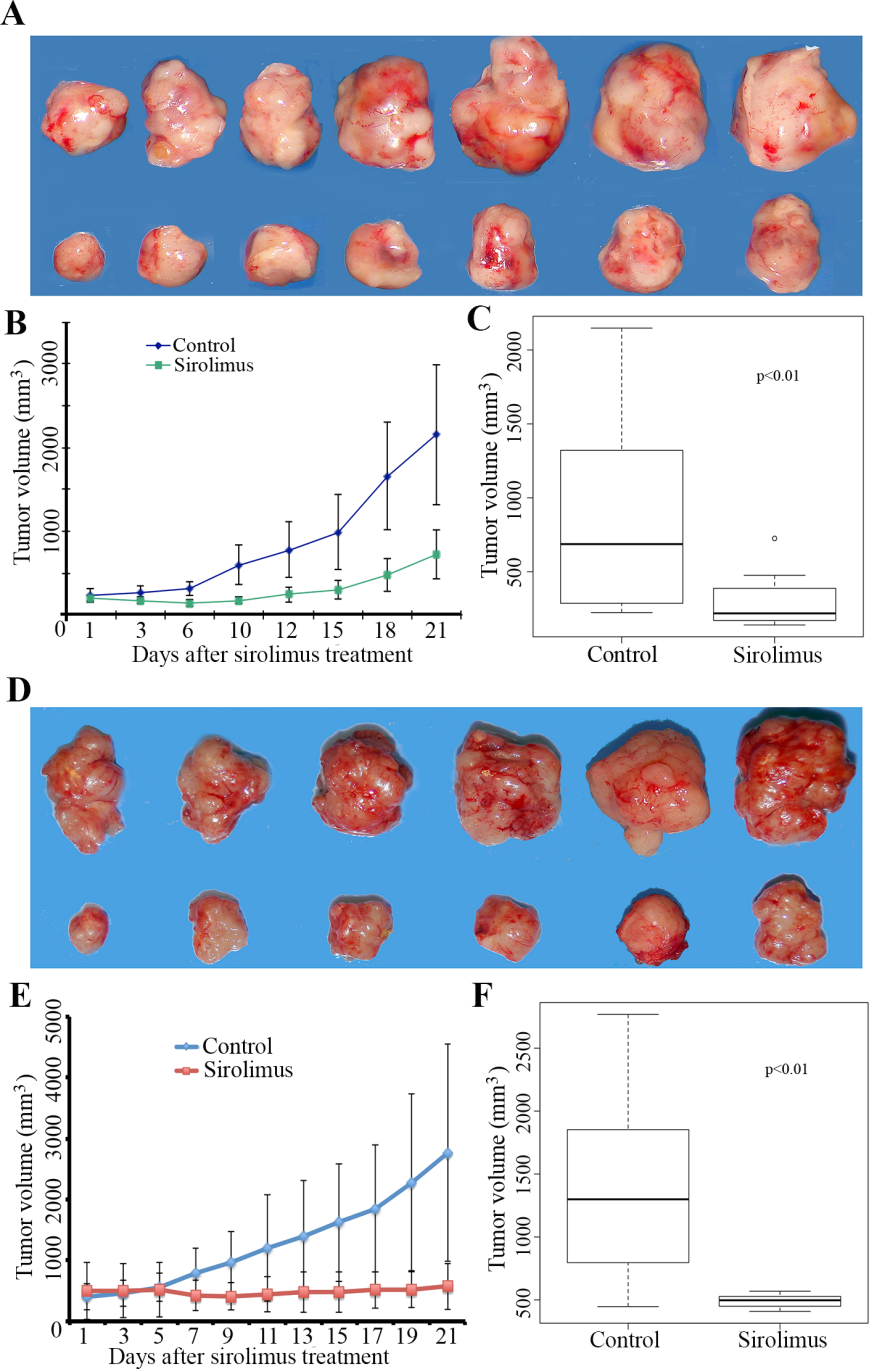
Sirolimus inhibits mouse allograft Flcn-deficient tumor growth **A.** representative tumors photographed from both sirolimus-treated mice (upper panel) and vehicle-treated control mice (lower panel). Sirolimus (7.5 mg/kg) was given every other day when allograft tumor size reached 200 mm^3^. **B.** growth curve of sirolimus-treated mice and vehicle-treated control mice. **C.** tumor growth was inhibited significantly by sirolimus compared to vehicle-treated controls (*p* < 0.01, *t*-test, showed in C). At the end of the treatment, tumors continued to grow slowly. **D.** representative tumors photographed from both sirolimus-treated mice (upper panel) and vehicle-treated control mice (lower panel). Sirolimus at a high dose (15 mg/kg) was given to treat the allograft tumors when the tumor size reached 450 mm^3^. **E.** growth curves of sirolimus-treated mice with high dosage of sirolimus treatment (15 mg/kg) and vehicle-treated control mice. **F.** tumor growth was inhibited significantly by sirolimus compared to vehicle-treated controls (*p* < 0.01, showed in F). Note that tumor growth had essentially stopped when treated with a higher dose of sirolimus.

To examine whether mTOR inhibitor sirolimus could suppress mouse Flcn-deficient tumor growth, we treated the above allograft-bearing nude mice with sirolimus at a dose of 7.5 mg/kg every other day. The treatment started when the allograft tumor reached ~200 mm^3^, and ended at the 21^st^ day. The average size of sirolimus-treated tumors increased to ~700 mm^3^ while the vehicle-treated control increased to ~2200 mm^3^ (Figure 4A–4B). The results indicated that allograft tumor growth was much slower in the sirolimus-treated group compared to the vehicle treated controls, suggesting that sirolimus could significantly inhibit the growth of these allograft tumors (Figure 4C).

To further determine whether a higher dosage of sirolimus would exhibit a dose dependent drug effect, we repeated the experiment by increasing the sirolimus dose to 15 mg/kg. Sirolimus was given to the mice when the allograft reached ~450 mm^3^. We found that sirolimus showed a strong suppressive effect on allograft tumor growth even when the treatment started later (~450 mm^3^ instead of ~200 mm^3^) (*p* < 0.05) (Figure 4D–4F). At the end of treatment, while the average tumor size in the control group grew to ~2700 mm^3^, the average size of sirolimus-treated tumors was ~570 mm^3^, a slight increase from ~450 mm^3^.

**Figure 5:**
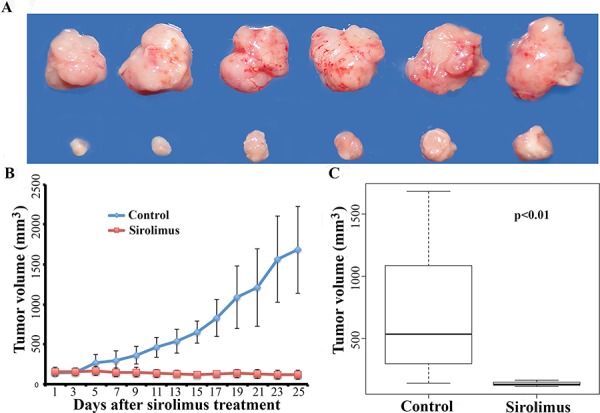
Sirolimus inhibits tumor growth **A.** representative tumors photographed from both sirolimus-treated mice (upper panel) and vehicle-treated control mice (lower panel). **B, C.** sirolimus (7.5 mg/kg) was given to the treatment group when allograft tumor size reached 150 mm^3^. Tumor growth was inhibited significantly by sirolimus compared to vehicle-treated controls (C). At the end of the treatment, allograft tumors shrank slightly.

**Figure 6:**
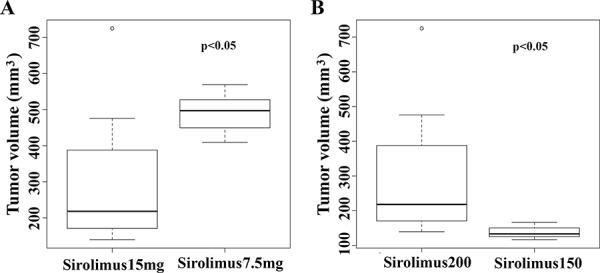
High dosages of sirolimus and early treatment lead to better treatment effects on tumor **A.** high dosage of sirolimus (15 mg/kg) exerted stronger inhibition on allograft tumor growth compared with low dose (7.5 mg/kg) (*p* < 0.05). **B.** sirolimus exerted more efficient suppression on smaller tumors at an early-stage (tumor size, <150 mm^3^).

To determine whether early-treatment of sirolimus could be more effective, we further performed another experiment by starting the sirolimus treatment when the allograft tumors only reached ~150 mm^3^ at a dose of 7.5 mg/kg. While the vehicle-treated control tumors grew fast, the sirolimus-treated tumors stopped growing and shrank (Figure 5A–5B). At the end of the treatment, sirolimus reduced the average tumor size to ~117 mm^3^ from average size of ~150 mm^3^, whereas the control tumors reached up to a size of ~2000 mm^3^. Clearly, both high-dose of sirolimus and early-treatment delivered better efficacy (*p* < 0.05, Figure 5C). Based on the above experiments, a high dosage (15 mg/kg) of sirolimus and early-treatment (tumor size ~150 mm^3^) could lead to more efficient tumor growth suppression in the allograft mouse model. The differences are statistically significant (Figure 6A–6B).

### Sirolimus suppressed xenograft tumor growth of human FLCN-deficient UOK 257-1 cells

**Figure 7:**
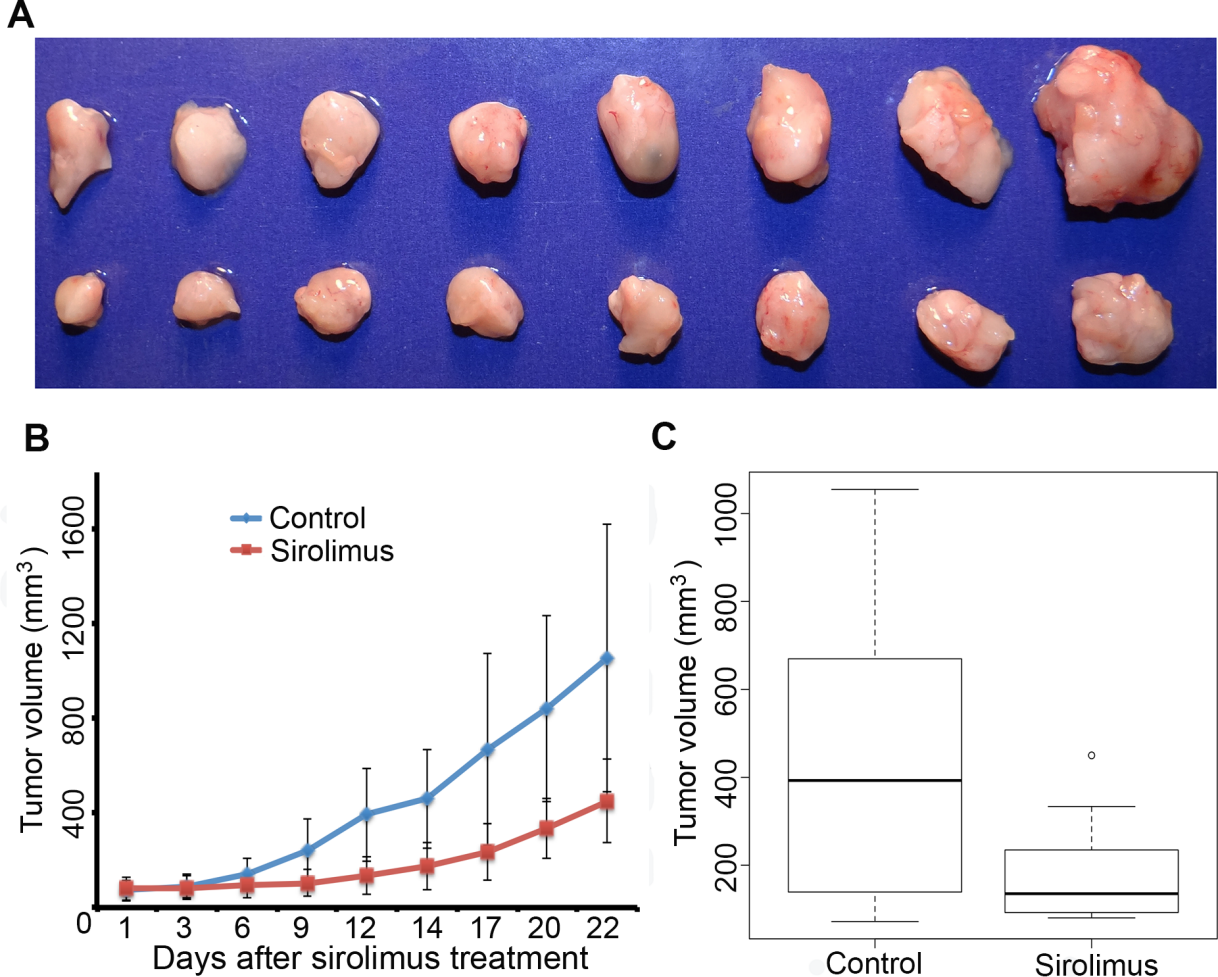
Sirolimus inhibits human UOK 257-1 xenograft tumor growth **A.** representative tumors photographed from both sirolimus-treated mice (upper panel) and vehicle-treated control mice (lower panel) at the end of the experiments (day 22). Sirolimus (15 mg/kg) was given every other day for 21 days when allograft tumor size reached 200 mm^3^. **B.** growth curves of sirolimus-treated mice and vehicle-treated control mice. **C.** at the end of treatment, tumors were significantly smaller in sirolimus-treated group compared to vehicle-treated controls (*p* < 0.05).

After the above results were obtained, we became interested in comparing the mouse allograft model with a human xenograft tumor model. The UOK 257 cell line is the only well-characterized RCC cell line that was derived from a BHD patient. To further test the efficacy of sirolimus on human FLCN-deficient RCC growth *in vivo*, we developed a xenograft mouse model by inoculating human FLCN-deficient RCC line, UOK 257-1, a cell line derived from a human BHD renal tumor [35]. It has been demonstrated that loss of the FLCN leads to the activation of the mTOR pathway in this cell line. Targeting mTOR by its inhibitor sirolimus is expected to suppress the growth of xenograft tumors developed from this cell line. When the tumor size reached 200 mm^3^, sirolimus was given to these mice at a dose of 15 mg/kg every other day. The results demonstrated that sirolimus caused tumor growth inhibition (Figure 7), which is consistent with the results obtained in the allograft tumors though the efficacy was slightly lower compared to the results obtained from the mouse allograft tumors.

## DISCUSSION

In this study, we first isolated the mouse Flcn-deficient cells from our previously developed knockout mouse *Flcn*^flox/flox^/*Ksp-Cre* that can only survive three weeks after birth due to the development of polycystic kidneys and blood urea nitrogen (BUN). We further demonstrated that these Flcn-deficient cells could evolve into tumorigenic cells and develop allograft tumors in athymic nude mice. The ability of the cells to develop into allograft tumors implies that deficiency of Flcn in kidney distal tubule-collecting duct-Henle's loop would lead to kidney cancer if the *Flcn*^flox/flox^/*Ksp-Cre* mice could survive longer. Our *in vitro* culture process might have played a significant role in “natural” selection of these premalignant or malignant cells. To our knowledge, this is the first time that by harvesting potential tumorigenic cells from an organ-specific tumor suppressor knockout mouse model and culturing these cells *in vitro* to overcome the shorter life expectancy of the knockout mouse model, the tumorigenic capability of cells with a tumor suppressor gene disruption was able to be tested. Otherwise, these cells would not have lived long enough *in vivo* to generate tumors.

The mouse allograft tumors also displayed the features of sarcomatoid RCC, indicating that loss of Flcn in renal distal tubule cells could result in highly aggressive tumors from these cells. Similar to renal cysts developed from our *Flcn*^flox/flox^/*Ksp-Cre* mice, these allograft tumors also exhibited the activation of the mTOR pathway, which is consistent with the previous findings in FLCN-deficient cell lines and animal models. To test whether mTOR inhibitors, such as sirolimus, could suppress tumor growth in allograft mice as well, we treated the allograft mice with various concentrations of sirolimus. Our results demonstrated that sirolimus could effectively inhibit Flcn-deficient allograft tumor growth, suggesting that mTOR inhibitors may be effective in treating FLCN-deficient RCC. Additionally, we demonstrated that a higher dose of sirolimus and earlier treatment delivered a better suppressive effect on tumor growth, showing a dose-dependent manner in tumor suppression.

To determine whether sirolimus can also exert suppression on human FLCN-deficient tumor growth, we developed a xenograft tumor mouse model for testing by inoculating human FLCN-deficient UOK 257-1 cells that were isolated from the original UOK 257 line [35]. Similarly, treatment of the UOK 257-1 xenograft with sirolimus led to suppression of tumor growth. Interestingly, it appeared that sirolimus was somewhat less effective against human UOK 257-1 xenograft tumor growth at a dose of 15 mg/kg compared to mouse allograft tumors. After 12 days treatment, the sirolimus-treated tumors started to grow, though much slower than the control group, suggesting that the tumors might have gradually developed resistance to sirolimus. In addition, the genetic background of the human UOK 257-1 cell line is probably different from the mouse allograft tumors, potentially carrying more DNA mutations, which may lead to the activation of other signaling pathways besides mTOR. Nonetheless, significant tumor suppression was observed in sirolimus-treated UOK 257-1 xenograft tumors. Thus, we provided first evidence that the mTOR inhibitor, sirolimus, can efficiently suppress both allograft and xenograft FLCN-dificient tumor growth.

Since the role of mTOR in cell proliferation and survival has been a major focus of research in human oncogenesis [44], and many genes associated with tumor-prone syndromes (e.g. hamartoma syndromes) are involved in the mTOR pathway [45], there has been considerable effort in developing mTOR-targeting drugs for use in oncology. In the case of kidney cancer, a sirolimus analog, temsirolimus, was approved for treating advanced stage RCC in 2007 based on a Phase III study and has been proven to be effective against RCCs that were primarily clear cell subtype [46]. The clinical benefit of temsirolimus for patients was demonstrated in the study comparing temsirolimus with interferon alpha (IFN-alpha) or combined temsirolimus plus IFN-alpha as first-line treatment of advanced RCC, showing treatment with temsirolimus alone significantly increased median overall survival in poor-risk, advanced RCC patients (10.9 vs 7.3 vs 8.4 months) [46]. To test whether temsirolimus is effective on other RCC subtypes, in another clinical study, temsirolimus was used for treating patients with non clear-cell and sarcomatoid variant ccRCC subtypes. The results indicated that the benefit from mTOR inhibitor was limited [47]. For the BHD-specific kidney tumorigenesis, the therapeutic approach could be different due to its specific genetic background and histological characteristics [14]. For example, while most of the kidney cancer-related genes are subtype-specific, FLCN mutations lead to all subtypes of RCCs that have been observed in BHD syndrome patients and knockout mouse models [10, 34, 48]. Thus, FLCN-deficient renal tumorigenesis might be associated with more than one pathway. In this study, we demonstrated that mTOR inhibitor, sirolimus, is able to suppress FLCN-deficient allograft and xenograft tumor growth. Currently, therapeutic approaches for BHD renal tumorigenesis and other mTOR-related FLCN-deficient neoplasia have not yet been described. Our findings could provide insights into the therapeutic strategy for BHD renal tumorigenesis and other FLCN-mTOR related tumor growth.

## MATERIALS AND METHODS

### Distal tubule-collecting duct-Henle's loop-specific knockout mice

The generation of distal tubule-collecting duct-Henle's loop-specific mice has been described in our previous study [31]. Briefly, *Flcn^flox/flox^* mice were first bred to *Ksp-Cre* transgenic mice to generate kidney-specific *Flcn* heterozygous knockout mice, *Flcn*^flox/+^/*Ksp-Cre*. These *Flcn*^flox/+^/*Ksp-Cre* heterozygotes were then backcrossed to *Flcn^flox/flox^* mice to generate kidney-specific homozygous knockout mice *Flcn*^flox/flox^/*Ksp-Cre*. The knockout mice developed polycystic kidneys with hyperplasia and early stage tumors (micro-tumors) [24]. However, the affected mice die from uremia within three weeks after birth and are not amenable to drug testing. To establish a Flcn-deficient mouse renal tumor model for drug study, cells from renal cysts and micro tumors of the affected *Flcn*^flox/flox^/*Ksp-Cre* kidneys were isolated and cultured in DMEM medium as described below.

### Isolation and culture of Flcn-deficient renal distal tubule cells

Six affected *Flcn*^flox/flox^/*Ksp-Cre* mice at age 20 days were used for isolation of Flcn-deficient renal distal tubule cells. Six cystic kidney tissues were aseptically excised from polycystic kidneys and each of them was transferred to a cell culture dish with DMEM medium, respectively. The tissues were then rinsed in sterile PBS. Fatty tissues, blood clots, and connective tissues were removed with forceps and scissors. Cleaned cystic tissues were finely minced into very small pieces in 0.25% trypsin-EDTA with sterile scissors. Tissue pieces were disassociated into small aggregates by vigorous pipetting. Heavier pieces were removed through sedimentation by gravity. Supernatant containing cell aggregates was transferred into a 15-ml centrifuge tube and centrifuged for 10 min at 1000 rpm. Cells were washed twice in PBS and transferred into a 25-cm^2^ flask and cultured in DMEM medium with 15% FBS. These cells were incubated at 37°C with 5% CO_2_. Medium was changed every 3 days. The cells were cultured for 35 passages.

### Genotyping

*Flcn*^flox/flox^/*Ksp-Cre* cells were harvested by scraping and collected in a 15-ml tube. After centrifugation, the cell pellet was resuspended in a solution of SDS detergent and proteinase K, and the mixture was incubated at 55°C for one hour. The sample was then phenol extracted once with a phenol/chloroform/isoamyl alcohol solution, and the aqueous layer was removed to a fresh microcentrifuge tube after centrifugation. The DNA was ethanol precipitated, resuspended in buffer, and then ethanol precipitated a second time. Once the pellet was dried, buffer was added and the DNA was re-suspended by incubation at 55°C overnight, after which the genomic DNA solution was assayed by the polymerase chain reaction (PCR). Genotyping was performed by PCR according to the previous report [31].

### Protein analysis

Whole-cell extracts were also prepared from these cells by lysing in 1% Nonidet P-40, 50 mM Tris (pH 7.4), 150 mM NaCl, 1 mM EDTA, and 15% glycerol, including standard protease inhibitors. Protein extracts were size separated by 8% SDS-PAGE and transferred to PVDF membranes. For the analysis of Flcn expression, Flcn was detected with an anti-FLCN monoclonal antibody at a dilution of 1:500 and an enhanced chemiluminescence detection system.

### Transplantation of Flcn-deficient renal cells into athymic nude mouse

Mouse Flcn-deficient renal distal tubule cells were maintained in DMEM medium with 10% FBS. Cells were harvested after 35 passages. Cells were washed with PBS and counted using a hemocytometer. Approximately 5× 10^6^ cells in 0.2 ml HBSS were transplanted subcutaneously into the flank area of each athymic nude mouse (B6.Cg-*Foxn1^nu^*/J, The Jackson Laboratory, USA). In the same fashion, approximately 5 × 10^6^ fast-growing human FLCN-deficient UOK 257-1 cells that were isolated from the original UOK 257 (gift from NCI) [35] were inoculated subcutaneously into each of 30 athymic nude mice to generate a human FLCN-deficient xenograft model. Fifteen mice were used for each experimental group. Same amount of HEK-293 normal kidney cells were inoculated into nude mice as control.

### Sirolimus treatment of allograft and xenograft mice

For each treatment experiment, 30 allograft or 30 xenograft athymic nude mice were equally randomized to one treatment group and one control group, with 15 mice in each group. Treatment commenced when the mice reached two months of age and tumors reached required sizes (150 mm^3^, 200 mm^3^, and 500 mm^3^). Sirolimus was formulated in a water solution containing 5% PEG-400, 4% ethanol, and 5% Tween-80. Each mouse in the treatment group received 7.5, 10, or 15 mg/kg body-weight sirolimus in 100 μl solution every other day through intraperitoneal injection, depending on treatment group. Mice in the vehicle control group were injected with 100 μl vehicle solution (5% PEG-400, 4% ethanol, and 5% Tween-80) each. All the mice were euthanized at the end of treatment.

### Immunohistochemistry

The antibodies used included mouse anti-FLCN mAb (N-terminal peptide: PQGDGNEDSPGQGEQC, produced at the Van Andel Research Institute), anti-Phospho-AKT Rabbit mAb (Cell Signaling), anti-Phospho-mTOR Rabbit mAb (Cell Signaling), anti-Phospho-S6 Ribosomal Protein (Cell Signaling). AQP1 (sc-25287, Santa Cruz Biotechnology); PAX2 (2549-1, Epitomics); CK7 (ab9021, Abcam; sc-80021, Santa Cruz Biotechnology) to determine RCC subtypes. Immunohistochemical analysis was performed following the manufacturers' protocols.
